# Supplementary material for: Integrated Proteomics and Metabolomic Analyses of Plasma Injury Biomarkers in a Serious Brain Trauma Model in Rats
Source: Int J Mol Sci. 2019 Feb 20;20(4):922. doi: 10.3390/ijms20040922 (PMC6412711; doi:10.3390/ijms20040922)
Supplement: Supplementary file 1 [file ijms-20-00922-s001.zip › ijms-435167-supplementary-2/Supplementary Files/Supporting Information.docx]

**Table S1.** Differentially changed metabolites in the plasma of the 1 day group identified by UPLC-Q-TOF/MS.

| **No.** | **Metabolite** | **Retention Time (min)** | **Ion (m/z)** | **VIP ^a^** | ***p*-Value ^b^** | **Fold Change ^c^** | **ESI Mode** |
| --- | --- | --- | --- | --- | --- | --- | --- |
| 1 | glutamine | 1.92 | 144.0672 | 1.37 | 0.001 | 2.78 | ESI- |
| 2 | 6-Fluoro-DL-try | 14.02 | 221.1486 | 1.53 | < 0.001 | 1.99 | ESI- |
| 3 | 4-Hydroxybenzaldehyde | 12.4 | 121.0299 | 1.38 | < 0.001 | -1.76 | ESI- |
| 4 | 7',8'-Dihydro-8'-hydroxycitraniaxanthin | 12.36 | 487.3204 | 1.10 | 0.023 | 5.54 | ESI- |
| 5 | 6beta-Hydroxyasiatic acid | 9 | 503.3351 | 1.15 | 0.006 | 2.22 | ESI- |
| 6 | phytolaccinic acid | 3.89 | 515.3389 | 1.08 | 0.013 | -1.56 | ESI- |
| 7 | pyridoxamine | 3.65 | 169.0969 | 1.15 | 0.002 | -2.03 | ESI+ |
| 8 | arginine | 1.91 | 175.0235 | 1.30 | < 0.001 | -3.13 | ESI+ |
| 9 | melatonin | 4.83 | 233.1579 | 1.33 | < 0.001 | -2.25 | ESI+ |
| 10 | phosphocholine | 7.57 | 440.2375 | 1.28 | < 0.001 | -3.8 | ESI+ |
| 11 | acetone | 12.74 | 59.049 | 1.21 | < 0.001 | 1.36 | ESI+ |
| 12 | 3-Aminopropionaldehyde | 14.97 | 74.0601 | 1.23 | < 0.001 | 1.10 | ESI+ |
| 13 | PC (14:0/18:1(11Z)) | 9.38 | 732.5486 | 1.10 | 0.005 | 1.31 | ESI+ |
| 14 | PC (22:2(13Z,16Z)/16:1(9Z)) | 13.42 | 812.6094 | 1.33 | < 0.001 | 6.97 | ESI+ |
| 15 | PC (22:6(4Z,7Z,10Z,13Z,16Z,19Z)/18:1(9Z)) | 9.4 | 832.5829 | 1.07 | 0.003 | 5.06 | ESI+ |

^a^ VIP was obtained from OPLS-DA with a threshold of 1.0.

^b^ P-value were evaluated by two-tailed Student’s t-test.

^c^ Fold change (injury/control) was calculated from the arithmetic mean values of two groups. The positive value indicate higher levels in injury group, and negative values indicate lower levels in injury group.

**Table S2.** Differentially changed metabolites in the plasma of the 3 day group identified by UPLC-Q-TOF/MS.

| **No.** | **Metabolite** | **Retention Time (min)** | **Ion (m/z)** | **VIP^a^** | ***p*-Value^b^** | **Fold Change^c^** | **ESI Mode** |
| --- | --- | --- | --- | --- | --- | --- | --- |
| 1 | 2-Phenyl-1,3-propanediol monocarbamate | 7.34 | 194.0816 | 1.70 | < 0.001 | -1.91 | ESI- |
| 2 | 2-[(3,3-dimethyloxiran-2-yl)methyl]-4-(3-methylbut-2-en-1-yl)benzene-1,3,5-triol | 8.06 | 277.1477 | 1.21 | 0.031 | -1.27 | ESI- |
| 3 | stearic acid | 11.32 | 283.2604 | 1.67 | 0.032 | -2.03 | ESI- |
| 4 | allolithocholic acid | 10.41 | 375.2809 | 1.24 | 0.002 | -1.47 | ESI- |
| 5 | arachidonoyl Serinol | 10.41 | 376.2845 | 1.94 | 0.001 | -1.77 | ESI- |
| 6 | lysoPE(0:0/16:0) | 8.08 | 452.2850 | 1.74 | 0.002 | -1.77 | ESI- |
| 7 | agavoside A | 8.13 | 591.3499 | 1.24 | 0.02 | -2.06 | ESI- |
| 8 | 4-Hydroxybenzaldehyde | 11.55 | 121.0295 | 1.25 | 0.016 | -1.87 | ESI- |
| 9 | 1-(6Z,9Z,12Z-octadecatrienoyl)-glycero-3-phosphate | 7.47 | 433.2419 | 2.28 | < 0.001 | -16.09 | ESI+ |
| 10 | 4-O-Methylmelleolide | 7.48 | 415.2119 | 2.27 | < 0.001 | -12.88 | ESI+ |
| 11 | 18-Hydroxy-5Z,8Z,11Z,14Z-eicosatetraenoic acid | 7.11 | 321.2429 | 1.91 | 0.001 | 1.38 | ESI+ |
| 12 | PC(14:0/20:0) | 11.40 | 762.5915 | 1.88 | 0.001 | 2.11 | ESI+ |
| 13 | hovenidulcigenin A | 8.92 | 545.3440 | 1.61 | 0.009 | 7.46 | ESI+ |
| 14 | cinncassiol D2 glucoside | 10.49 | 531.2751 | 1.49 | 0.017 | -1.94 | ESI+ |
| 15 | PC(22:5(7Z,10Z,13Z,16Z,19Z)/P-16:0) | 9.89 | 792.5948 | 1.48 | 0.018 | 1.67 | ESI+ |
| 16 | scutigeral | 4.10 | 373.2331 | 1.43 | 0.024 | 1.19 | ESI+ |
| 17 | imidazolone | 12.71 | 85.0393 | 1.38 | 0.043 | 2.21 | ESI+ |
| 18 | hydrogen phosphate | 13.56 | 98.9745 | 1.31 | 0.041 | 1.20 | ESI+ |
| 19 | acetone | 5.02 | 59.0495 | 1.51 | 0.001 | 1.81 | ESI+ |
| 20 | octadecanamide | 11.96 | 284.2950 | 1.28 | 0.017 | 2.85 | ESI+ |
| 21 | 12alpha-hydroxy-3-oxo-5beta-cholan-24-oic Acid | 4.24 | 391.2845 | 1.24 | 0.002 | -3.35 | ESI+ |

^a^ VIP was obtained from OPLS-DA with a threshold of 1.0.

^b^ P-value were evaluated by two-tailed Student’s t-test.

^c^ Fold change (injury/control) was calculated from the arithmetic mean values of two groups. The positive value indicate higher levels in injury group, and negative values indicate lower levels in injury group.

**Table S3.** The stability and reproducibility of the UPLC-Q-TOF/MS analytical system was assessed by eight ions of the QC samples in both positive and negative ESI mode.

| **Peaks No.** | **ESI+** | | |  | **ESI-** | | |
| --- | --- | --- | --- | --- | --- | --- | --- |
|  | **Retention time (min)** | **m/z** | **RSD (%)** |  | **Retention time (min)** | **m/z** | **RSD (%)** |
| 1 | 8.591 | 81.0694 | 2.27 |  | 8.938 | 481.3223 | 4.24 |
| 2 | 9.149 | 579.2926 | 3.18 |  | 10.933 | 827.5765 | 3.58 |
| 3 | 13.333 | 717.5558 | 5.24 |  | 12.919 | 351.2562 | 5.08 |
| 4 | 14.315 | 356.0697 | 3.12 |  | 13.15 | 514.3307 | 4.26 |
| 5 | 14.968 | 61.0393 | 5.53 |  | 3.09 | 61.9891 | 1.16 |
| 6 | 9.822 | 448.338 | 3.27 |  | 7.22 | 174.9558 | 5.64 |
| 7 | 6.069 | 87.0432 | 5.24 |  | 8.84 | 165.0418 | 3.74 |
| 8 | 8.135 | 239.0881 | 3.27 |  | 9.72 | 502.3774 | 4.71 |

**Table S4.** Differentially expressed proteins in plasma of the injury group identified by the iTRAQ-based quantitative proteomics approach.

| **No.** | **Protein Name** | **Gene Name** | **Uniprot ID** | **Unique Peptides (95%)** | **% Cov (95%)** | **Unused** | **Fold Change (p-values)** | |
| --- | --- | --- | --- | --- | --- | --- | --- | --- |
|  |  |  |  |  |  |  | **1 day** | **3 day** |
| 1 | Serum albumin | Alb | P02770 | 4 | 80.76000213 | 178.02 | 0.14 (0.02) | 0.03 (0.005) |
| 2 | Alpha-1-inhibitor 3 | A1i3 | P14046 | 19 | 41.90999865 | 114.99 | 0.06 (0.000) | 0.03 (0.000) |
| 3 | LOC367586 protein | LOC367586 | Q5M7V3 | 23 | 71.14999890 | 75.64 | 0.23 (0.000) | 0.33 (0.002) |
| 4 | Alpha-2-macroglobulin | A2m | P06238 | 29 | 26.55999958 | 47.21 | 0.06 (0.000) | 0.04 (0.001) |
| 5 | T-kininogen 2 | T-kininogen 2 | P08932 | 10 | 57.66999721 | 45.75 | 0.09 (0.000) | 0.04 (0.000) |
| 6 | Group specific component | Gc | Q68FY4 | 20 | 54.40999865 | 40.24 | 0.08 (0.000) | 0.03 (0.000) |
| 7 | Alpha-1-antiproteinase | Serpina1 | A0A0G2JZ73 | 21 | 53.97999882 | 39.09 | 0.45 (0.000) | 0.37 (0.000) |
| 8 | Murinoglobulin-1 | Mug1 | Q03626 | 20 | 42.42999851 | 37.83 | 0.06 (0.000) | 0.03 (0.000) |
| 9 | Afamin | Afm | G3V9R9 | 18 | 37.5 | 35.51 | 0.05 (0.000) | 0.07 (0.000) |
| 10 | Alpha-2-HS-glycoprotein | Ahsg | P24090 | 17 | 51.99000239 | 32.47 | 0.07 (0.000) | 0.74 (0.254) |
| 11 | Kallikrein B, plasma 1 | Klkb1 | Q5FVS2 | 16 | 36.52000129 | 31.58 | 0.52 (0.152) | 1.92 (0.038) |
| 12 | Inter-alpha trypsin inhibitor, heavy chain 1 | Itih1 | B2RYM3 | 14 | 23.89000058 | 26.98 | 0.15 (0.046) | 0.27 (0.011) |
| 13 | Histidine-rich glycoprotein | Hrg | A0A0G2K3G0 | 15 | 29.85000014 | 26.57 | 0.37 (0.000) | 0.11 (0.000) |
| 14 | Similar to RIKEN cDNA 1300017J02 | RGD1310507 | A0A0G2K896 | 15 | 29.28999960 | 26.46 | 0.1 (0.000) | 0.04 (0.000) |
| 15 | Ig gamma-2B chain C region | Igh-1a | P20761 | 14 | 48.64999949 | 24.49 | 0.12 (0.006) | 1.62 (0.441) |
| 16 | Inter-alpha-trypsin inhibitor heavy chain H3 | Itih3 | D3ZBS2 | 12 | 17.81000047 | 21.96 | 0.26 (0.003) | 0.18 (0.000) |
| 17 | Carboxylic ester hydrolase | Ces1c | D3ZGK7 | 10 | 23.11999946 | 20 | 0.07 (0.001) | 0.03 (0.006) |
| 18 | Globin a4 | Hbb | A0A0G2JSW3 | 5 | 71.42999768 | 18.07 | 0.04 (0.001) | 0.04 (0.05) |
| 19 | Fetub protein | Fetub | Q6IRS6 | 9 | 36.39000058 | 17.75 | 0.11 (0.02) | 0.07 (0.002) |
| 20 | Inter-alpha-trypsin inhibitor heavy chain 2 | Itih2 | D3ZFH5 | 11 | 21.54999971 | 17.19 | 0.1 (0.001) | 0.22 (0.000) |
| 21 | Transthyretin | Ttr | P02767 | 7 | 62.58999705 | 14.15 | 0.28 (0.056) | 0.06 (0.004) |
| 22 | Globin c3 | Hba-a1 | B1H216 | 7 | 58.45000147 | 13.97 | 0.14 (0.023) | 0.05 (0.000) |
| 23 | Kininogen 1 | Kng2 | Q5PQU1 | 7 | 48.14000129 | 12 | 0.13 (0.001) | 0.03 (0.000) |
| 24 | Angiotensinogen | Agt | P01015 | 6 | 14.88000005 | 11.12 | 0.41 (0.018) | 0.05 (0.000) |
| 25 | Ig kappa chain C region, A allele | N/A | P01836 | 7 | 81.12999796 | 10.12 | 0.7 (0.08) | 0.44 (0.023) |
| 26 | Complement factor I | Cfi | A0A0G2K135 | 6 | 15.50000011 | 9.94 | 0.52 (0.152) | 0.03 (0.011) |
| 27 | Alpha-1-acid glycoprotein | AGP | A0A0H2UHF8 | 4 | 22.13000059 | 7.17 | 0.13 (0.04) | 0.02 (0.006) |
| 28 | Globin a2 | LOC689064 | A0A1K0FUA6 | 4 | 70.06999850 | 7.16 | 0.14 (0.04) | 0.04 (0.032) |
| 29 | Corticosteroid-binding globulin | Serpina6 | P31211 | 3 | 6.818000227 | 5.74 | 0.08 (0.022) | 0.07 (0.008) |
| 30 | Haptoglobin | Hp | A0A0H2UHM3 | 22 | 59.24999713 | 43.07 | 0.08 (0.1) | 0.04 (0.000) |
| 31 | RCG33981, isoform CRA_a | Serpinf2 | Q68FT8 | 11 | 22.81000018 | 22.03 | 0.12 (0.002) | 0.63 (0.28) |
| 32 | Hemopexin | Hpx | P20059 | 29 | 52.82999873 | 55.17 | 0.07 (0.000) | 0.03 (0.000) |
| 33 | Plasma protease C1 inhibitor | Serping1 | Q6P734 | 14 | 37.09999918 | 27.83 | 0.29 (0.009) | 1.05 (0.787) |
| 34 | Tubulin beta-5 chain | Tubb5 | P69897 | 4 | 41.44000113 | 6 | 0.08 (0.008) | 0.75 (0.622) |
| 35 | Glyceraldehyde-3-phosphate dehydrogenase | Gapdh | P04797 | 13 | 42.64000058 | 25.27 | 0.15 (0.000) | 0.23 (0.009) |
| 36 | C4b-binding protein alpha chain | C4bpa | Q63514 | 4 | 72.21999764 | 123.66 | 2.61 (0.035) | 0.96 (0.311) |
| 37 | Prosaposin | Psap | Q6P7A4 | 16 | 36.34999990 | 29.92 | 0.42 (0.125) | 1.89 (0.004) |
| 38 | Ribonuclease A b1 | Rnase4 | W0UVG1 | 7 | 55.77999949 | 14 | 1.53 (0.172) | 2.99 (0.03) |
| 39 | Apolipoprotein C-III | Apoc3 | A0A0G2K8Q1 | 10 | 77.99999713 | 12.89 | 1.25 (0.54) | 2.31 (0.048) |
| 40 | Fibrillin 1 | Fbn1 | G3V9M6 | 9 | 4.978999868 | 11.76 | 3.73 (0.008) | 0.62 (0.749) |
| 41 | Apolipoprotein N | Apon | Q5M890 | 6 | 34.50999855 | 11.21 | 2.47 (0.119) | 7.94 (0.001) |
| 42 | Glycosylphosphatidylinositol specific phospholipase D1 | Gpld1 | G3V8B1 | 29 | 45.19999921 | 51.49 | 1.79 (0.006) | 3.91 (0.000) |
| 43 | Lumican | Lum | P51886 | 7 | 24.56000000 | 14.18 | 0.29 (0.478) | 2.01 (0.035) |

**
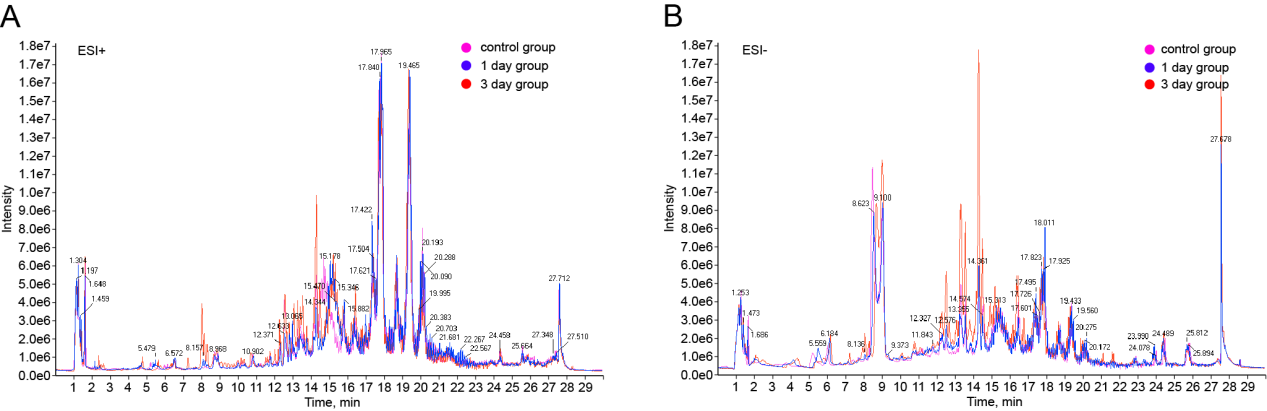
**

**Figure S1.** Typical TIC chromatograms of plasma samples from the control and injury. groups. A: ESI+; B: ESI-.


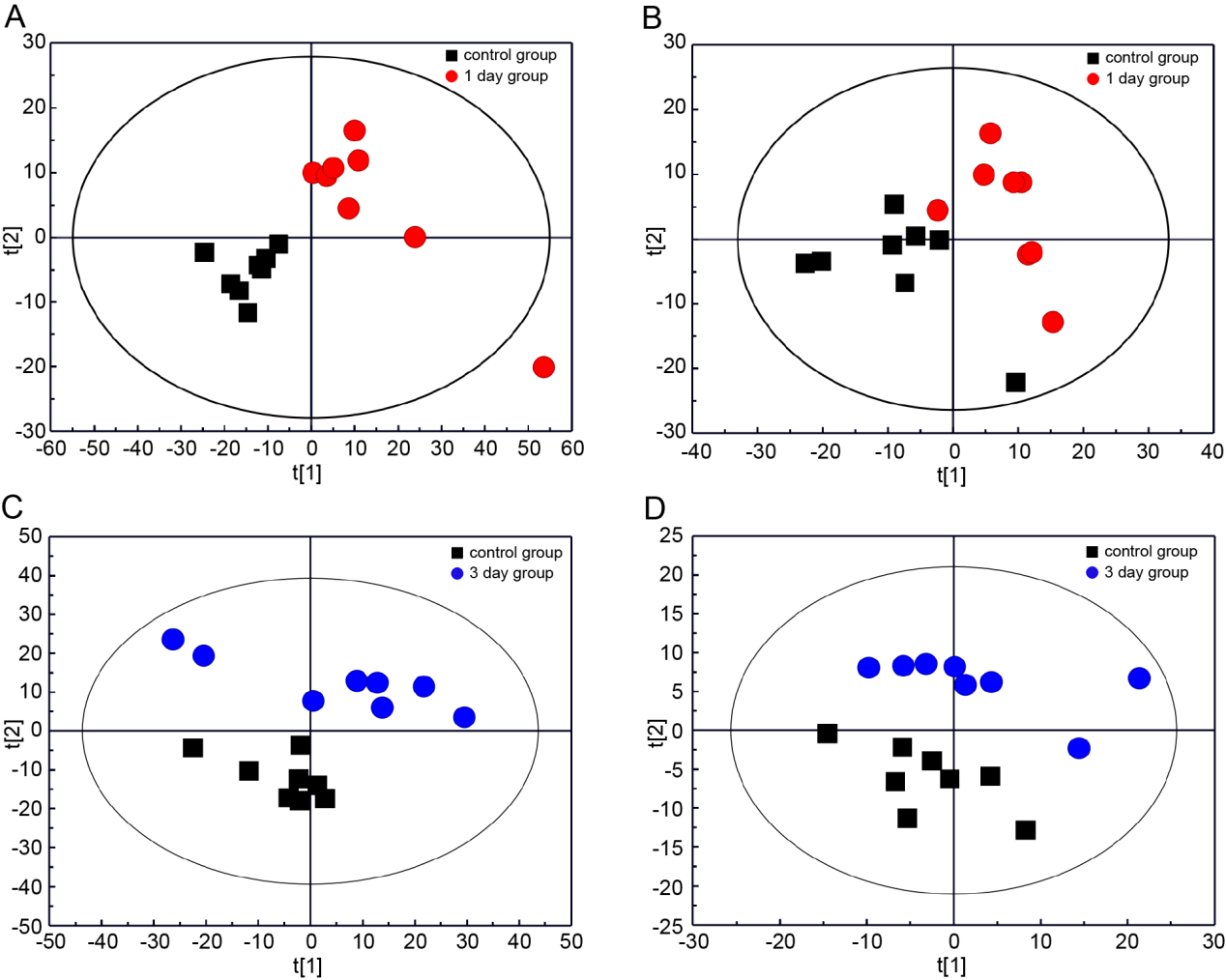


**Figure S2.** Score plots for PCA analysis of UPLC-Q-TOF/MS-based metabonomic data from the controls (black rectangles), 1 day subjects (red dots) and 3 day subjects (blue dots).


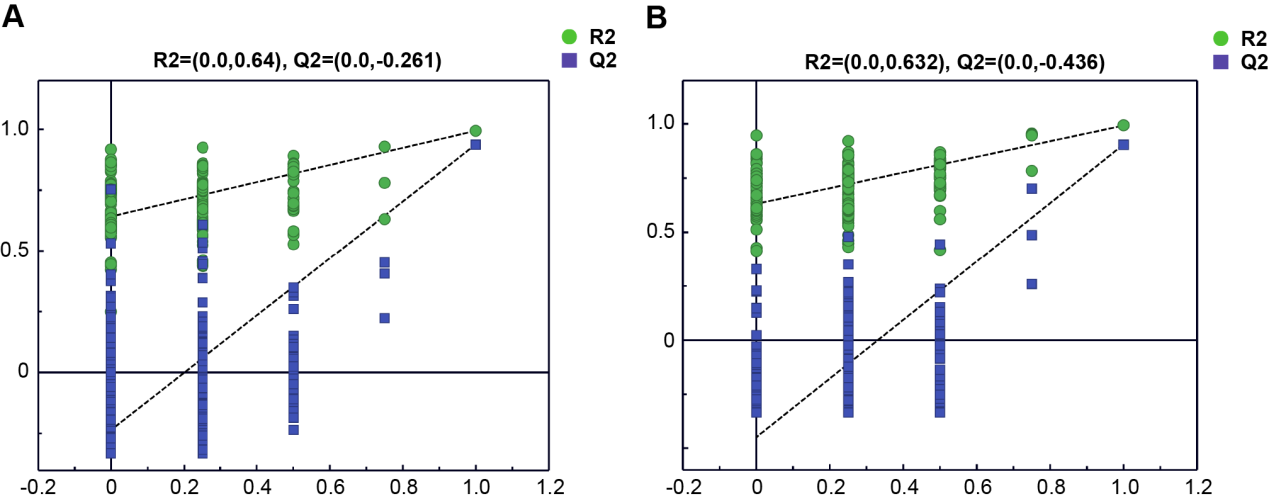


**Figure S3.** Cross-validation plot of OPLS-DA mode of UPLC-Q-TOF/MS-based metabonomic data from the controls and 1 day subjects with 300 times permutation tests. A: ESI+; B: ESI-.


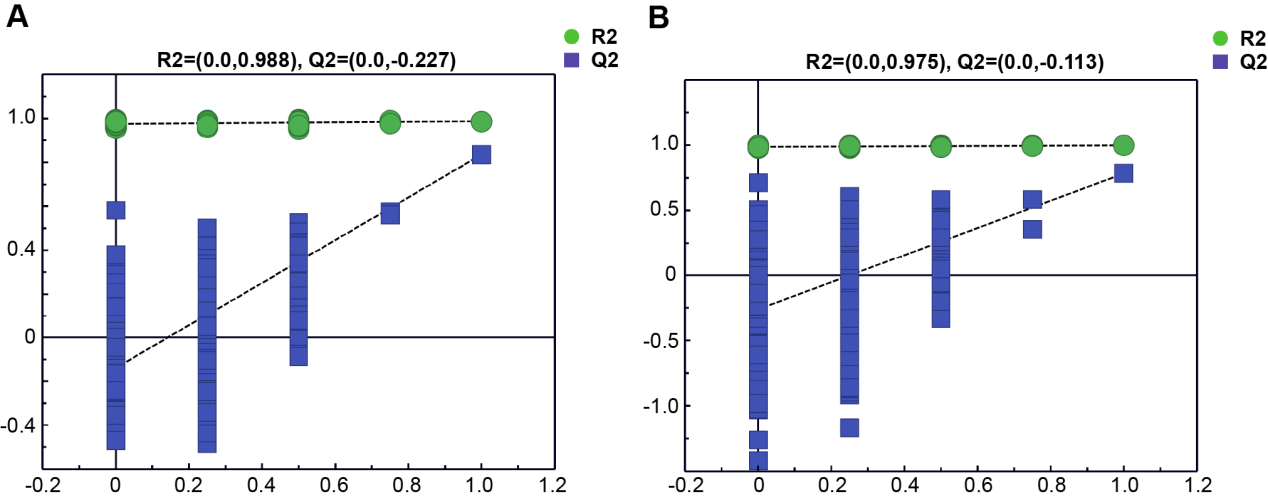


**Figure S4.** Cross-validation plot of OPLS-DA mode of UPLC-Q-TOF/MS-based metabonomic data from the controls and 3 day subjects with 300 times permutation tests. A: ESI+; B: ESI-.

**
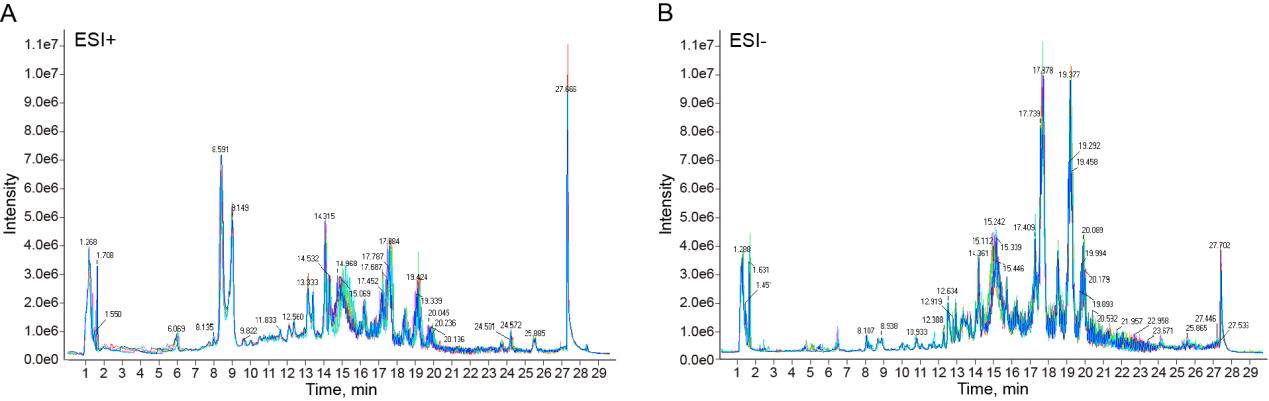
Figure S5.** The overlaps of the spectral peak of six within-run QC samples in positive (A) and negative (B) ESI mode.
